# Lifespan Extension Induced by Caffeine in *Caenorhabditis elegans* is Partially Dependent on Adenosine Signaling

**DOI:** 10.3389/fnagi.2015.00220

**Published:** 2015-12-08

**Authors:** Jessika Cristina Bridi, Alexandre Guimarães de Almeida Barros, Letícia Reis Sampaio, Júlia Castro Damásio Ferreira, Felix Alexandre Antunes Soares, Marco Aurélio Romano-Silva

**Affiliations:** ^1^Departamento de Saúde Mental, Faculdade de Medicina, Universidade Federal de Minas GeraisBelo Horizonte, Brazil; ^2^Instituto Nacional de Ciência e Tecnologia de Medicina Molecular, Faculdade de Medicina, Universidade Federal de Minas GeraisBelo Horizonte, Brazil; ^3^Departamento de Bioquímica e Biologia Molecular, Centro de CiênciasNaturais e Exatas, Universidade Federal de Santa MariaSanta Maria, Brazil

**Keywords:** caffeine, lifespan, adenosine, insulin/IGF-1 pathway, *Caenorhabditis elegans*

## Abstract

Caffeine is a widely used psychoactive substance. Studies have shown that caffeine may play a protective role in aging-associated disorders. However, the mechanisms by which caffeine modulates aging are not yet clear. In this study, we have shown that caffeine increases *Caenorhabditis elegans* lifespan, delays its larval development, reduces reproduction and body length. These phenotypes were partly reversed by worm’s exposure to adenosine, which suggest a putative common target. Moreover, they were dependent on a functional insulin/IGF-1-like pathway. Our results may shed light on new genetic determinants of aging.

## Introduction

Caffeine is a psychoactive substance used worldwide (Fredholm, 2012). It is present in many ordinary products like coffee, tea, soft drinks, chocolate and found in over-the-counter pills (Fredholm et al., 1999; Fisone et al., 2004). Studies have projected that 70% of the adult population consumes quantities of caffeine capable of affecting their behavior on a daily basis (Fredholm, 2012).

Pharmacodynamics of caffeine has been ascribed mainly to its nonselective antagonism of adenosine receptors (Chen et al., 1999; Fredholm et al., 1999). At low levels, caffeine blocks all four humans’ adenosine receptors (A_1_, A_2A_, A_2B_ and A_3_; Chen et al., 1999). Most of its effects appears to be dependent on interactions with A_1_ and A_2A_ receptors and to a lesser extent, with A_2B_ and A_3_ receptors (Chen et al., 1999; Fredholm et al., 1999). At higher concentrations, caffeine has looser interactions and may disturb calcium release from intracellular stores, inhibit GABAA receptors, the enzymes 5′-nucleotidase and alkaline phosphatase (Chen et al., 1999; Fredholm et al., 1999).

Studies suggest that chronic caffeine exposure may be beneficial in many neuropsychiatric disorders like Alzheimer’s and Parkinson’s diseases (Souza et al., 2003; Ritchie et al., 2007) due to slow cognitive decline promoted by caffeine (Cunha and Agostinho, 2010). For instance, mice exposed to caffeine show better cognitive test results and reduced levels of β-amyloid in their brains (Dall’Igna et al., 2007; Cao et al., 2009). Caffeine also seems to prevent avoidance memory impairment in rats (Gevaerd et al., 2001). In worms, caffeine reduces the level of β-amyloid aggregation in transgenic strains expressing the peptide in muscle cells and partly reverses induced-paralyzes (Dostal et al., 2010). In humans, caffeine intake was inversely related to chronic diseases such as diabetes, inflammatory diseases, and stroke (Freedman et al., 2012). However, caffeine intake has also been suggested to cause side effects such as fetal growth restriction, late miscarriage, increased risk of osteoporosis, and anxiety (Lara, 2010; Fitt et al., 2013). In spite of its presence on daily-life and ascribed potential as a modulator of aging, the molecular mechanisms through which caffeine interacts remains largely unclear.

*Caenorhabditis elegans* (*C. elegans*) has emerged as one of the most powerful systems to study aging (Klass, 1977; Hsin and Kenyon, 1999; Corsi, 2006). Studies have uncovered many genes interfering with the aging process in worms. Usually the ascribed function of these genes are related to stress-response pathways and energy homeostasis (Kenyon, 2010b). For instance, the gene *daf-2* codes for the worms’ insulin receptor orthologue and function in response to changes in food availability and stress situations (Kenyon et al., 1993; Murphy et al., 2003). Also, studies have shown that loss-of-function mutations in *daf-2* increases worms’ lifespan (Kenyon et al., 1993; Kimura et al., 1997). This is partly because the disruption of DAF-2 releases the transcription factor DAF-16 to the nucleus allowing for increased expression of many genes related to resilience (Kimura et al., 1997; Hsu et al., 2003; Samuelson et al., 2007; Tullet et al., 2008; Kenyon, 2010b). In addition to DAF-16, other transcription factors have their function modified by DAF-2, such as SKN-1, HSF-1 and PQN-1 (Kimura et al., 1997; Hsu et al., 2003; Samuelson et al., 2007; Kenyon, 2010b; Bansal et al., 2014).

Given the high levels of caffeine’s consumption worldwide and its potential as a determinant of a healthy aging (Freedman et al., 2012), we aimed to evaluate whether and how caffeine influences the aging process using the nematode *C. elegans*. We found that caffeine was able to extend worm’s lifespan. In addition, worm’s increased lifespan was reversed by concurrent exposure to adenosine, which suggests a common target pathway. Furthermore, disruption of the insulin/IGF-1-like pathway was able to lessen lifespan extension induced by caffeine.

## Materials and Methods

### Strains and Culture Conditions

*C. elegans* strains were cultured as described before (NGM). The *E. coli* OP50 was used as food source (Brenner, 1974). N2 Bristol was used as wild-type. The mutant strains used in this study were: *daf-16 (mu86)* I outcrossed 12× to our wild-type, *daf-2 (e1370) III* outcrossed 4X to our wild-type and TJ356 *(Is[daf-16P::daf-16::GFP; rol-6(su1006)])*. All strains were obtained from the Caenofrhabditis Genetics Center. For all experiments, synchronized L1 populations were acquired through hypochlorite treatment of gravid adults (Barros et al., 2014).

### Caffeine and Adenosine Treatments

Caffeine and adenosine (Sigma-Aldrich) were freshly dissolved in deionized water and added into NGM after autoclaving. Plates were prepared the day before use.

### Lifespan Assay

Lifespan assays were performed on NGM plates at 20°C (Kenyon et al., 1993; Lin et al., 2001; Berman and Kenyon, 2006). L1 worms (day 0) were transferred to NGM plates supplemented with caffeine and/or adenosine and/or vehicle for different periods of time as stated in the figure legends. Worms were scored every day or every other day, and those that failed to respond to a gentle prodding with a platinum wire were scored as dead (Li et al., 2008). Animals were transferred to new plates every other day until they ceased laying eggs. After the end of the reproductive period, worms were moved onto new plates once per week.

### Development Assay

Worm developmental stage was evaluated every day until they reached complete development (gravid adults). They were scored as L1, L2, L3 or L4 larval stages, young adult (no eggs in uterus) or adult (gravid adults) following body morphology criteria (Altun and Hall, 2012).

### Body Length

Body length was calculated by measuring the tip-to-tail length of individual adult animals. Digital images were taken using a stereomicroscope fitted with a camera and length measured with NIS-Element AR 3 software (Nikon) (Gubert et al., 2013).

### Brood Size Assays

Each worm was allowed to lay eggs and transferred to fresh NGM plate every 24 h until the egg laying period was finished. The number of hatched worms was counted after 48 h of incubation at 20°C (Li et al., 2008) . Worms that crawled off the plate, exploded or bagged were censored (Hsu et al., 2009). Twelve worms were used for each treatment condition. The experiment was repeated at least three times.

### DAF-16 Localization Assay

DAF-16::GFP animals* (Is[daf-16P::daf-16::GFP; rol-6(su1006)])* were cultured at 20°C and imaged at L4 stage. Worms were exposed to caffeine for different periods of time (30 min, 1, 2 and 48 h). Worms were mounted on slides with an agarose pad and paralyzed with azide. Images were taken using a confocal microscope system (Leica TCS SP5) with Leica Application Suite Advanced Fluorescence (LAS AF). The relation between fluorescence signal in nucleus and cytoplasm was quantified using Image-J software (NIH). The average fluorescence from six different cells per worm were calculated and used for analyzes (Driver et al., 2013). Each experiment was repeated at least three times and 12–17 worms per group were randomly selected in each experiment.

### Statistical Analysis

Statistical analysis was performed using GraphPad Instat (Version 5.0 for Macintosh OSX, GraphPad Software, San Diego, CA, USA). The Kaplan-Meier method was applied to calculate survival fractions and log-rank (Mantel-Cox) test was used to compare survival curves. The Bonferroni’s post-test correction was applied for multiple comparisons. Student’s *t*-test or one-way Analysis of Variance (ANOVA) followed by Bonferroni post-test was used to check for significant differences between means for other comparisons. *P*-values lower than 0.05 were considered significant.

## Results

### Lower Concentration of Caffeine Increases Worm’s Lifespan

**Figure 1:**
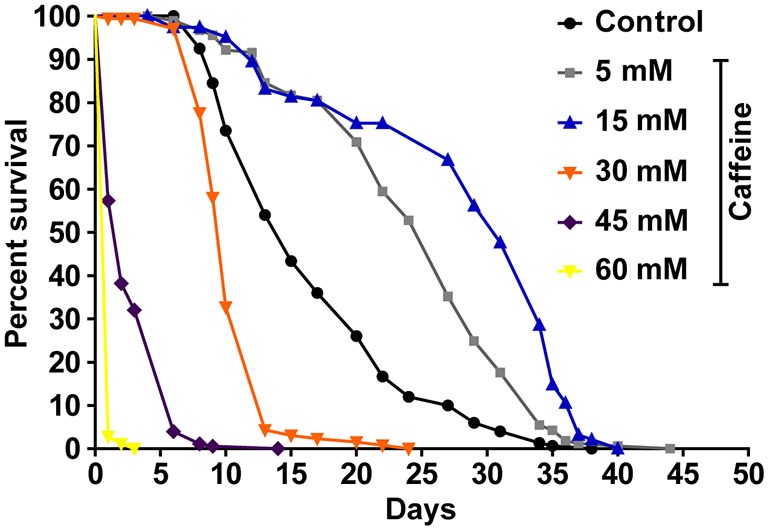
**Exposure to caffeine increases lifespan of wild-type worms.** Survival curves of wild-type animals exposed to caffeine. Caffeine’s treatment results in lifespan extension at 5 and 15 mM. At higher concentrations (30, 45 and 60 mM), caffeine reduces worm’s life expectancy. *p* < 0.0001 for each condition compared to control. The experiment was performed at least three times. Additional information see Table 1.

To determine whether caffeine extends worm’s lifespan, we exposed L1 wild-type worms to different caffeine concentrations and measured how long they lived (Figure 1 and Table 1). Caffeine increased worm’s lifespan in lower concentrations while it exhibited an opposite effect at higher concentrations. These results suggest that caffeine has a dual role on worm’s lifespan and may point to a toxic effect at higher concentrations.

**Table 1:** **Lifespan of wild-type worms exposed to caffeine**.

Genotype	Caffeine (mM)	Median lifespan (days)	Number of animals (n)/censored	*P* value vs. control
Wild-type	–	15	189/33
	5	27	189/22	<0.0001
	15	31	194/87	<0.0001
	30	10	176/14	<0.0001
	45	2	178/0	<0.0001
	60	1	181/0	<0.0001

### Caffeine Delays Larval Development

**Figure 2:**
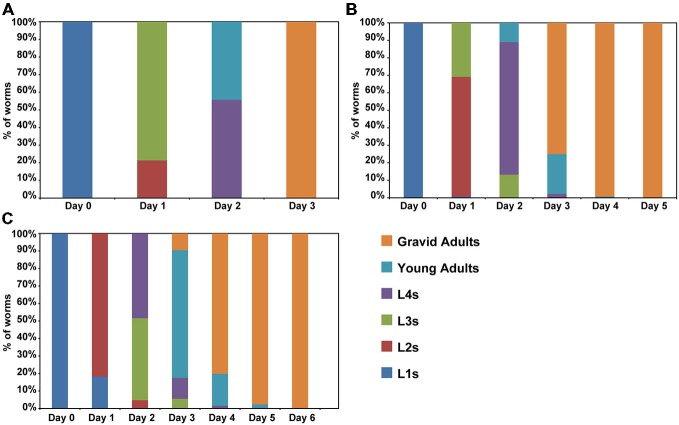
**Caffeine disrupts normal larval development. (A–C)** Caffeine delays larval development. L1 wild-type worms exposed to caffeine require longer time to achieve adult stage. **(A)** Control worms reach adult stage within 3 days (*n* = 131 worms). **(B)** Worms exposed to 5 mM caffeine need 4 days to complete development (*n* = 136 worms). **(C)** Worms exposed to 15 mM caffeine exhibited a larger delay on larval development (*n* = 126 worms). The experiment was performed three times at 20°C.

Previous studies have suggested that aging is a process that starts early in life (Britton et al., 2008; Felix et al., 2013). For instance, premature stressful events in humans may have adverse outcomes later in life (Wahlbeck et al., 2001; Caraci et al., 2010; Saczynski et al., 2010). In *C. elegans*, events during larval development also have effects on lifespan. Overcrowding, fasting and other adverse conditions are capable to active an alternative development pathway, called dauer that is remarkable resistant (Golden and Riddle, 1984). To determine whether caffeine exposure increases lifespan through disruption of larval development, worms were exposed to caffeine and their larval development evaluated until adulthood (Figure 2). Caffeine disrupted larval development. Wild-type animals achieved adult stage at the third day of life while worms exposed to caffeine delayed their larval development in a dose-dependent manner. Noteworthy, worms exposed to high concentrations of caffeine had an accentuated delay in their larval development and at the highest concentrations, did not develop beyond the first larval stage and died after a couple of days. This outcome suggests that caffeine is toxic at higher concentrations, as reported in the previous study (Min et al., 2015), in agreement with the reduction in lifespan of worms exposed to higher levels of it.

### Caffeine Induced Lifespan Extension Requires Lifelong Exposure

**Figure 3:**
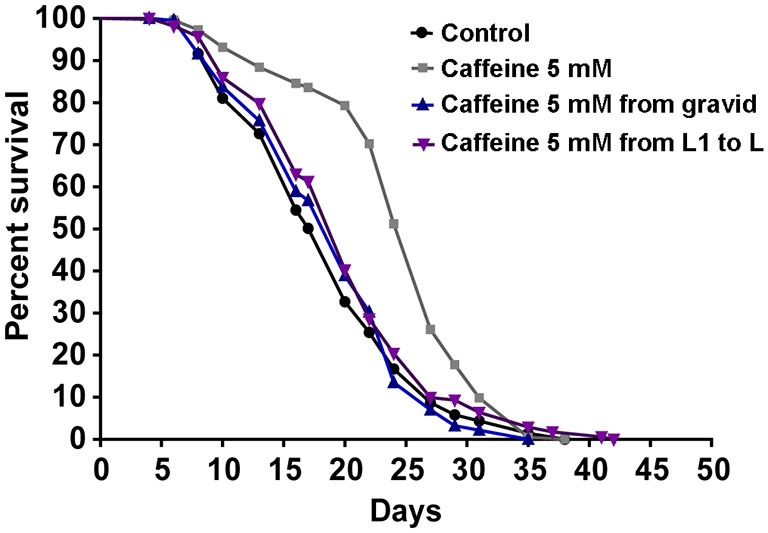
**Lifespan extension requires lifelong caffeine exposure.** Survival curves of wild-type worms exposed to 5 mM caffeine for different periods. Worms exposed to caffeine from L1 until L4 larval stage or from adult until death had lifespan similar to control animals, *p* = 0.0885 and *p* = 0.9157, respectively. Worms lifelong exposed to caffeine showed an increased lifespan compared to control group *p* < 0.0001. Experiment was performed three times. For additional information see Table 2.

Many studies suggest that lifespan is affected by numerous processes (Partridge and Gems, 2002; Kenyon, 2010b). Also, our results suggest that caffeine disrupts larval development process. With this in mind, one possible explanation for caffeine-induced increase in lifespan may be that the adult stage is reached later in animals exposed to caffeine. Additionally, it is possible that caffeine may interfere with lifespan-extending processes that take place during larval development. To verify whether caffeine’s lifespan extension results exclusively through its exposure during larval development, worms were treated with caffeine at different larval stages and lifespan measured (Figure 3 and Table 2). We chose 5 mM caffeine to perform further experiments because it increases lifespan and does not halt development. Worms showed similar lifespan to controls when exposed to caffeine only during larval development or starting after adult stage was reached. Therefore, caffeine’s lifespan extension requires lifelong exposure at 5 mM.

**Table 2:** **Lifespan of wild-type worms exposed to caffeine at different stage and time-period**.

Genotype	Caffeine (mM)	Treatment	Median lifespan (days)	Number of animals (n)/censored	*P* value vs. control
Wild-type	0	–	20	220/78	
	5	Entire lifetime	27	227/22	<0.0001
	5	Adult	20	228/41	0.9157
	5	L1–L4	20	224/48	0.0885

### Caffeine Reduces Worm’s Size and Egg Production

**Figure 4:**
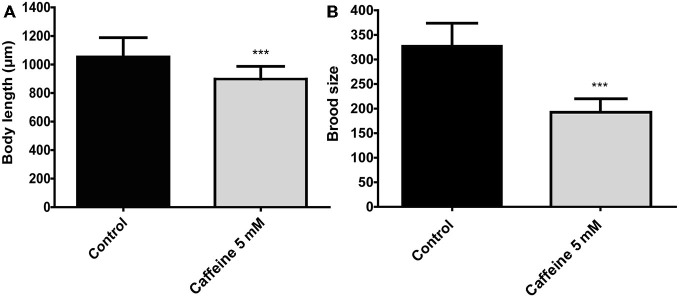
**Caffeine reduces size and reproduction in wild-type worms. (A,B)** Measurements of worm’s length and brood size. **(A)** Adult worms exposed to 5 mM caffeine from L1 larval stage have a reduced body length (top-to-tail) compared to control worms. The measurement was conducted when animals achieved adult stage. Black and gray bars represent control and caffeine exposed animals, respectively. Data are showed as mean ± SD. ***p < 0.0001 compared to control animals, *n* = 12 worms per group. **(B)** Wild-type worms exposed to 5 mM caffeine show a sharp reduction on egg-laying compared to control animals. Black and gray bars represent control and caffeine exposed animals. Data are shown as mean ± SD. ***p < 0.0001 compared to control animals, *n* = 22–40 worms.

Previous studies suggest that processes that require generous amounts of energy have a significant impact on lifespan. For instance, animals that reproduce less or have its energy stores consumed slowly shows an increase lifespan (Arantes-Oliveira et al., 2002). Therefore, caffeine may extend lifespan by interfering with aspects related to energy expenditure. To evaluate whether caffeine has influence on these process, growth and reproduction were measured (Figure 4). Caffeine exposure reduced the mean body length of adult worms. Assuming that increased energy is required to maintain larger worms, it is possible that reduced body length may influence, somehow, caffeine induced lifespan extension. However, the reduction in body size resulted from contact with caffeine during larval development and lifespan extension requires caffeine exposure throughout worm’s life. Caffeine also disrupted worm’s egg production. Similar to body’s length, egg production is an energy costly process (Mukhopadhyay and Tissenbaum, 2007). Moreover, pharyngeal pumping and defecation behavior were also assessed in worms exposed to caffeine, however, there were no significant difference compared to control group (data not shown). Our results point to multiple behaviors under influence of caffeine and suggest that caffeine’s induced lifespan extension is an outcome underpinned by them (López-Otín et al., 2013).

### Caffeine Induced Lifespan Extension Seems to be Partly Dependent on DAF-2 But Not on DAF-16

**Figure 5:**
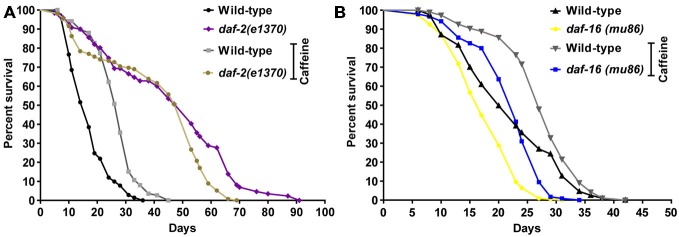
**Caffeine-induced lifespan extension is partly dependent on DAF-2 but not on DAF-16. (A,B)** Survival curves of *daf-2* and *daf-16* mutants lifelong exposed to caffeine. **(A)** *daf-2* mutants had a longer lifespan phenotype compared to wild type. *daf-2* mutants exposed to 5 mM caffeine had a slightly decrease in lifespan. *p* = 0.0005, *daf-2* control group compared to *daf-2* animals exposed to caffeine. **(B)** *daf-16* mutants exposed to 5 mM caffeine had an increase in lifespan similar to wild-type worms. *p* < 0.0001, *daf-16* control group compared to *daf-16* group exposed to caffeine. Experiment was performed three times. For additional information see Table 3.

In worms, the insulin/IGF-1 signaling pathway is a major molecular hub to mechanisms that influences lifespan (van Heemst, 2010). Disruption of the *daf-2* gene is linked to increases in worm’s lifespan and stress resistance. It has been ascribed to changes in the activity of many downstream targets that mainly acts as transcription factors. To investigate whether caffeine’s induced lifespan extension depends on insulin/IGF-1 pathway, loss-of-function mutants for both DAF-2 and DAF-16 were exposed to it and their lifespan evaluated (Figure 5 and Table 3). *daf-2* mutants exposed to caffeine showed a slight decrease in lifespan compared to vehicle-exposed controls while *daf-16* loss-of-function mutants showed an increased life expectancy similar to that observed for wild-type animals (Figure 5). Taken together, these results suggest that DAF-2 is necessary for caffeine lifespan extension. Conversely, the phenotype may result from the biologic viability extreme that *daf-2* animals experience and no further lifespan extension would be possible. However, the lifespan reduction observed when *daf-2* mutants were exposed to caffeine points to another direction. Our data shows caffeine may increase or decrease lifespan based on the treatment concentration employed. Therefore, the slightly lifespan reduction observed in *daf-2* mutants may represent the toxic effect of caffeine at lower concentration. This suggests that the increase in lifespan induced by caffeine may be ascribed to a functional insulin/IGF-1-like signaling pathway.

**Table 3:** **Lifespan of wild-type, *daf-2 (e1370)* and *daf-16 (mu86)* worms exposed to caffeine since L1**.

Genotype	Caffeine (mM)	Median lifespan (days)	Number of animals (n)/censored	*P* value vs. control
Wild-type	–	17	189/39	
	5	28	180/64	<0.0001
*daf-2 (e1370)*	–	53	121/18
	5	53	206/58	0.0005
Wild-type	–	20	216/18
	5	27	193/7	<0.0001
*daf-16 (mu86)*	–	17	227/7
	5	23	243/10	<0.0001

**Figure 6:**
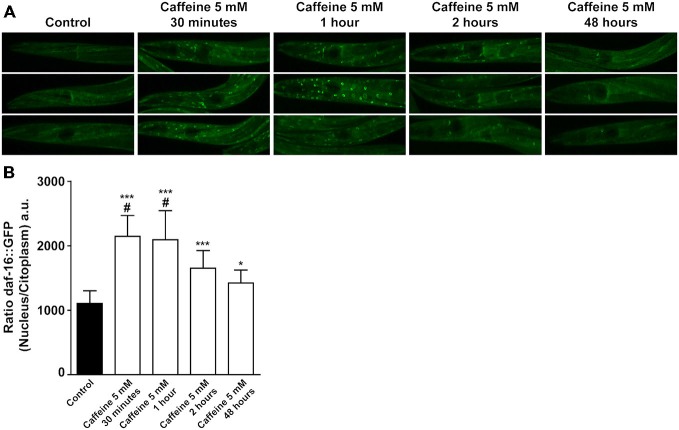
**Acute caffeine exposure translocates DAF-16 to nucleus. (A,B)** Caffeine’s exposition for a short period of time translocates DAF-16 from cytoplasm to nucleus. **(A)** Representative images of worms from the same experimental group and larval stage and **(B)** fluorescence quantification of daf-16::gfp animals exposed to 5 mM caffeine during different time points. Black bar and gray bars represent control and 5 mM caffeine exposure, respectively. Data are showed as mean ± SD from six random cells from each worm. *p < 0.05 and ***p < 0.0001, caffeine exposed animals compared to control animals. ^#^*p* < 0.0001 animals exposed to caffeine for 30 min and 1 h compared to 48 h exposed animals. *n* = 12–17 worms were evaluated in each group.

DAF-2 modulates downstream targets to ultimately phosphorylates DAF-16. Phosphorylated DAF-16 is not able to translocated from the cytoplasm to the nucleus, halting its transcriptional activity. To further explore how caffeine modulates the DAF-2 signaling pathway, a transgenic worm expressing DAF-16 tagged with a green fluorescent protein (GFP; *Is[daf-16P::daf-16::GFP; rol-6(su1006)])* was subjected to caffeine exposure and the subcellular location of DAF-16 analyzed over time. Animals exposed to caffeine from L1 to L4 larval stage showed a higher DAF-16::GFP nuclear/cytoplasm fluorescence ratio than vehicle-treated worms. Curiously, L4 worms exposed to caffeine for shorter periods of time exhibited a fluorescence ratio even higher (Figures 6A,B).

### Adenosine Antagonizes Caffeine-Induced Lifespan Extension and Brood Size Reduction

**Figure 7:**
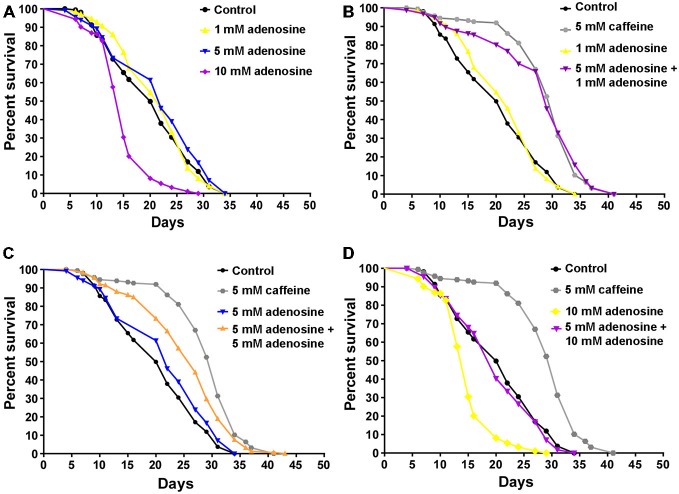
**Adenosine partially antagonizes caffeine’s lifespan increase. (A–D)** Survival curves of wild-type animals exposed to caffeine and/or adenosine. **(A)** Animals exposed to different concentrations of adenosine. 10 mM adenosine reduced worm’s lifespan. *p* < 0.0001, 10 mM adenosine exposed animals compared to control animals. **(B–D)** Adenosine partly reversed worm’s lifespan extension caffeine-induced. For *p*-values and *n* please see Table 4. Experiment was performed three times.

**Figure 8:**
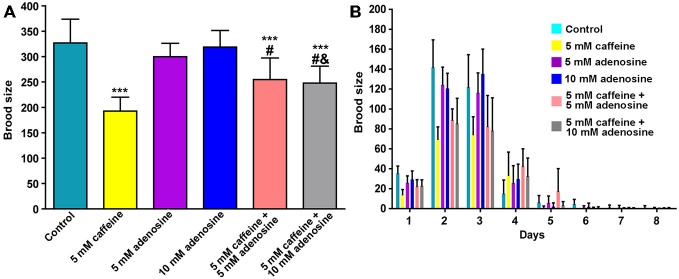
**Adenosine partially reverses caffeine’s brood size reduction. (A,B)** Quantitation of egg-laying phenotypes of animals exposed to caffeine and/or adenosine. **(A)** Adenosine partially reversed brood size reduction induced by caffeine. Each color represents one condition. Data are showed as mean of 12 animals per group ± SD. ***p < 0.0001 compared to control group. ^#^*p* < 0.001 compared to 5 mM caffeine group. ^&^*p* < 0.0001 compared to 10 mM adenosine group. **(B)** Distribution of offspring through fertile period of wild-type worms exposed to caffeine and/or adenosine.

In mammals, caffeine works as an adenosine receptor antagonist (Cunha and Agostinho, 2010). Therefore, caffeine might interact in worms with similar targets of those already known in mammals. To verify this hypothesis, wild-type worms were exposed to caffeine and/or adenosine and their lifespan measured. Worms exposed to adenosine had a significantly shorter median lifespan (Figure 7A and Table 4). Moreover, wild-type worms exposed to both adenosine and caffeine, adenosine was able to reverse caffeine-induced lifespan extension in a concentration-dependent manner (Figures 7B–D and Table 4). To gain insight about this interaction, worms also had their reproduction assessed after exposure to caffeine and/or adenosine (Figure 8). As expected, adenosine partly reversed caffeine-induced reduction in egg-laying.

**Table 4:** **Lifespan of wild-type worms exposed to caffeine and/ adenosine since L1**.

Genotype	Caffeine (mM)	Adenosine (mM)	Median lifespan (days)	Number of animals (n)/censored	*P* value vs. control	*P* value vs. 5C	*P* value vs. Adenosine
Wild-type	–	–	20	163/26
	5	–	31	174/17	<0.0001
	–	1	22	174/40	0.5304
	–	5	22	136/10	0.0847
	–	10	15	195/10	<0.0001
	5	1	29	162/15	<0.0001	0.8621	<0.0001
	5	5	27	187/25	<0.0001	0.0001	<0.0001
	5	10	20	163/42	0.3993	<0.0001	<0.0001

## Discussion

In this study, we demonstrated that caffeine increases lifespan of the nematode *C. elegans*. Caffeine also interfered with the worm’s reproduction, size and development. It is unclear whether these phenotypes result from the same molecular mechanism or from distinct pathways. Our data suggest possible pathways related to caffeine-induced phenotypes. We observed that DAF-2 signaling pathway is necessary for increase in lifespan induced by caffeine. Moreover, caffeine was able to modify DAF-16 traffic between nucleus and cytoplasm, supporting its modulatory role over this canonical pathway. Furthermore, caffeine may interact with targets similar to that in mammals. This is suggested by the reversal of caffeine-induced phenotypes observed with concurrently exposure to adenosine. It is well established that these molecules interact antagonistically on purinergic receptors and many of the effects of caffeine are mimicked by specific antagonists of adenosine receptors (Fisone et al., 2004; Cunha and Agostinho, 2010).

In recent years, the effects of caffeine on aging have been extensively studied (Cunha and Agostinho, 2010). This is because caffeine is one of the most widely consumed psychoactive substance worldwide (Fredholm et al., 1999). Caffeine intake has been associated with increased longevity in humans and animal models (Lublin et al., 2011; Freedman et al., 2012). However, the mechanisms underlying this phenotype are not well established. Caffeine-induced lifespan extension has been previously reported in worms (Lublin et al., 2011; Sutphin et al., 2012). A recent study has shown that caffeine’s increases lifespan of adult worms (Sutphin et al., 2012). We were not able to replicate this data. In our experimental conditions, worms had to be exposed to caffeine from L1 larval stage in order to observe increase in lifespan. Therefore, caffeine exposure has different outcomes dependent on the conditions, dose and length of exposure.

Aging is a complex process that starts early in life (Britton et al., 2008; Felix et al., 2013). Studies have shown that factors such as birth rate and stressful environments are important determinants of healthy aging and may result in adverse outcomes late in life (Wahlbeck et al., 2001; Colman et al., 2009; Caraci et al., 2010; Saczynski et al., 2010). One explanation is that energetic costly events like these may result in adverse conditions such as increased production of reactive oxygen species (Speakman, 2005). As stated before, worms’ larval development has remarkable effects on lifespan. Therefore, worm’s lifespan seems to result from processes that happen during the entirely life cycle. Another example is *clk* mutants worms, which are deficient in enzyme involved in the synthesis of ubiquinone. These worms are characterized by slow physiologic rates, delayed larval development, decreased brood size and increased lifespan (Hansen et al., 2005; Van Raamsdonk et al., 2010). This suggests that increased lifespan may be achieved by decreasing energy expenditure throughout life (Van Raamsdonk et al., 2010). In worms, reproduction, larval development and body size are energetic costly and may influence lifespan (Arantes-Oliveira et al., 2002; Jia et al., 2004; Mukhopadhyay and Tissenbaum, 2007). As a result, it is possible that the ability of caffeine to disrupt these processes may affect life expectancy as a whole.

In worms, many genes have been linked to increased longevity (Kenyon, 2010b). Insulin/IGF-1-like signaling is a well-established pathway that regulates aging. It regulates several cellular processes responsible for oxidative stress resistance, immunity and energy balance (Amrit and May, 2010). Loss-of-function mutations in *daf-2* increase lifespan in part by activation of the transcription factor DAF-16, a member of the FOXO family (Kimura et al., 1997; Kenyon, 2010a). Caffeine-induced lifespan extension was dependent on insulin/IGF-1 signaling pathway. *daf-2* mutants showed no increase in lifespan after exposure to caffeine. This suggests that caffeine may extend lifespan through downregulation of insulin/IGF-1-like signaling pathway. Remarkable, this mechanism is similar to that observed in long-lived *daf-2* mutants. In a short time-frame, caffeine exposure significantly increased DAF-16 levels in the nucleus. This further suggests that caffeine inhibits the insulin/IGF-1 pathway. However, DAF-16 was not essential for caffeine induced lifespan extension. In fact, studies have showed that DAF-16 transcriptional activity is not sufficient to extend lifespan. For example, worms bearing mutated DAF-16 that constitutively localizes in the nucleus have similar control’s lifespan (Lin et al., 2001; Berman and Kenyon, 2006). Therefore, caffeine-induced lifespan extension is partly dependent on insulin/IGF-1 signaling pathway but not through modulation of DAF-16 transcriptional factor. This is in disagreement with previously published studies (Lublin et al., 2011; Sutphin et al., 2012). They have showed that caffeine does not increased lifespan of *daf-16* mutant strains, thus, demonstrating that it is acting through DAF-16 to promote extension of longevity. These may result from different experimental conditions. For instance, we have used a *daf-16 (mu86)* strain that was outcrossed to our wild-type twelve times. This procedure has been shown to be crucial for experiments with this strain, possibly because of close linked mutations (Lin et al., 2001). Moreover, in our experiments worms were exposed to caffeine from the first larval stage to end of their lives while in previous studies exposure started in adult stage.

Even though insulin/IGF-1-like signaling pathway results ultimately in AKT-dependent phosphorylation of DAF-16, other transcriptional factors such as SKN-1 and HSF-1 (Murphy and Hu, 2013) could potentially be modulated by caffeine’s exposure and thereby, cooperating to this increase in lifespan.

Studies have showed that blockage of adenosine A_2A_ receptors by caffeine may attenuate damage caused by amyloid peptide. In addition, long-term caffeine administration protects Aβ-expressing transgenic mice against cognitive impairment. Furthermore, blockade of A_2A_ receptors has been shown to exert neuroprotective action during cerebral ischemia (Chen et al., 1999). We found that adenosine reduces lifespan extension induced by caffeine and partially recover the caffeine-induced decline in brood size. Taken together, our data suggest a common molecular target. This pharmacological approach sheds light on a participation of adenosine signaling in the extension of lifespan. This is supported by experiments in *Drosophila melanogaster*, where components of the purinergic system have been demonstrated to be involved in adult lifespan (Stenesen et al., 2013). In conclusion, the present study demonstrates that caffeine exhibits a remarkable influence over *C. elegans* lifespan. Further studies are necessary for further elucidation of the molecular mechanisms by which caffeine modulates lifespan. This may disclose new genetic determinants of aging.

## Conflict of Interest Statement

The authors declare that the research was conducted in the absence of any commercial or financial relationships that could be construed as a potential conflict of interest.
